# Oxylipins in Aqueous Humor of Primary Open-Angle Glaucoma Patients

**DOI:** 10.3390/biom14091127

**Published:** 2024-09-05

**Authors:** Jianming Xu, Kewen Zhou, Changzhen Fu, Chong-Bo Chen, Yaru Sun, Xin Wen, Luxi Yang, Tsz-Kin Ng, Qingping Liu, Mingzhi Zhang

**Affiliations:** 1 Joint Shantou International Eye Center of Shantou University and The Chinese University of Hong Kong, Shantou 515041, China; xjm@jsiec.org (J.X.); zkw@jsiec.org (K.Z.); fcz@jsiec.org (C.F.); ccb@jsiec.org (C.-B.C.); syr@jsiec.org (Y.S.); wenxin@jsiec.org (X.W.); ylx@jsiec.org (L.Y.); wjz@jsiec.org (T.-K.N.); 2 Shantou University Medical College, Shantou 515041, China; 3 Department of Ophthalmology and Visual Sciences, The Chinese University of Hong Kong, Hong Kong; 4 Guangdong-Hong Kong-Macao University Joint Research Laboratory of Precision Prevention and Treatment on Ocular Diseases, Shantou 515041, China; 5 Guangdong Provincial Engineering Technology Research Center for Precision Diagnosis and Treatment on Ocular Diseases of Guangdong Province, Shantou 515041, China

**Keywords:** primary open-angle glaucoma, aqueous humor, oxylipins, arachidonic acid

## Abstract

Purpose: Investigate the oxylipin profiles in the aqueous humor of primary open-angle glaucoma (POAG) patients. Methods: Aqueous humor samples were collected from 17 POAG patients and 15 cataract subjects and subjected to a liquid chromatography/mass spectrometry (LC-MS) analysis to detect the oxylipins. The prediction potential of the differential abundant oxylipins was assessed by the receiver operating characteristic (ROC) curves. Pathway and correlation analyses on the oxylipins and clinical and biochemical parameters were also conducted. Results: The LC-MS analysis detected a total of 76 oxylipins, of which 29 oxylipins reached the detection limit. The multivariate analysis identified five differential abundant oxylipins, 15-keto-prostaglandin F2 alpha (15-kPGF2α), Leukotriene B4 (LTB4), 12,13-Epoxyoctadecenoic acid (12,13-Epome), 15-Hydroxyeicosatetraenoic acid (15-HETE) and 11-Hydroxyeicosatetraenoic acid (11-HETE). The five oxylipins are enriched in the arachidonic acid metabolism and linoleic acid metabolism pathways. Pearson correlation analysis showed that 11-HETE was positively correlated with intraocular pressure and central corneal thickness and negatively with cup/disk area ratio in the POAG patients. In addition, 15-kPGF2α was moderately and positively correlated with the mean deviation (MD) of visual field defect, and LTB4 was moderately and negatively correlated with macular thickness. Conclusions: This study revealed the oxylipin profile in the aqueous humor of POAG patients. Oxylipins involved in the arachidonic acid metabolism pathway could play a role in POAG, and anti-inflammatory therapies could be potential treatment strategies for POAG.

## 1. Introduction

Glaucoma is a leading cause of irreversible visual impairment and blindness, with 111.8 million individuals expected to be affected by 2040 [1]. Primary open-angle glaucoma (POAG), a common subtype of glaucoma, is characterized by visual field (VF) defects and the progressive loss of retinal ganglion cells (RGCs) [2,3]. Current POAG treatments are limited to lowering IOP treatments [1,2]. However, a prospective cohort study [4] reported that 42 out of 179 eyes (23.5%) with well-controlled IOP exhibit continuously progressive visual field loss over a five-year follow-up period, suggesting that additional risk factors could be involved in the development of POAG.

Dysregulation in lipids has been implicated in the development of POAG [5,6]. We previously identified the association of the *CAV1* and *ABCA1* variants, the genes for lipid transfer, with POAG [7]. Other genome-wide association studies (GWASs) have identified lipid genes associated with POAG, such as LIPC, CDKN2B, and GAS7 [6,8]. The ABCA1 gene regulates cholesterol in the eye through high-density lipoprotein (HDL) and reverse cholesterol transport [9]. The Singapore Epidemiology of Eye Disease study, involving 175 POAG patients, found a significant reduction in HDL-3, suggesting that disrupted reverse cholesterol transport is important in POAG pathogenesis [6]. Zeleznik et al. analyzed plasma metabolites in 599 glaucoma patients from three U.S. databases and validated in the UK Biobank database. All cohorts showed that higher glyceride levels are adversely associated with glaucoma, suggesting a significant role of lipids in glaucoma [10]. Other studies suggest that elevated triglycerides may increase blood viscosity, which could raise episcleral venous pressure and IOP [11].

Based on previous studies, we further identified that the POAG patients have significantly higher plasma triglycerides but lower high-density lipoprotein (HDL) cholesterol levels than the cataract subjects. Moreover, we identified that the low-density lipoprotein (LDL) subclass, LDL3, small dense LDL, and oxidized LDL(ox-LDL) were significantly higher in the POAG patients with elevated total cholesterol and/or LDL-cholesterol levels [12]. We also demonstrated that ox-LDL can promote the expression of pro-inflammatory cytokines and increase the levels of fatty acid and sphingomyelin metabolites in microglia and macrophages [12]. Critically, we recently revealed that multiple oxylipins, 15-keto-prostaglandin F2 alpha, 13,14-dihydro-15-keto-prostaglandin D2, 11-dehydro-thromboxane B2, 8,9-epoxyeicosatrienoic acid, and arachidonic acid are significantly decreased in the plasma samples of POAG patients [13].

Oxylipins are a significant component of lipid metabolites, and our previous studies have highlighted their importance in the plasma of POAG patients [7,12,13]. Aqueous humor is known to more directly reflect intraocular metabolic states [14]. However, the oxylipins profile in the aqueous humor of POAG patients has not yet been characterized.

This study aimed to determine the profiles of oxylipins in the aqueous humor samples of the POAG patients. The metabolic pathways, the correlation with clinical and biochemical parameters, and the prediction potential of the oxylipins were also evaluated.

## 2. Materials and Methods

### 2.1. Study Subjects

In total, 17 POAG patients and 15 cataract control subjects were enrolled. This study’s protocol has been approved by the Ethics Committee for Human Medical Research at the Joint Shantou International Eye Center of Shantou University and the Chinese University of Hong Kong, which is in accordance with the tenets of the Declaration of Helsinki (IRB numbers: EC 20210313(2)-P03). Written informed consent was obtained from all study subjects after explaining the nature and possible consequences of this study. Currently, there is no established formula for sample size calculation in targeted metabolomics. The sample size in this study was determined based on previously published articles.

The inclusion criteria of the POAG subjects included IOP > 21 mmHg at diagnosis, open anterior chamber angle by gonioscopy, cup-to-disk (C/D) area ratio > 0.5 or binocular C/D area ratio differences > 0.2, retinal nerve fiber layer (RNFL) thinning, and mean deviation (MD) visual field defects of ≤20% for false negative rate and false positive rate and ≤33% for fixation loss rate. The patients with secondary glaucoma and previous glaucoma surgery were not included in this study. Age- and sex-matched senile cataract control subjects without glaucoma and other eye diseases were recruited.

The demographic data and the results of the blood test were retrieved from the electronic medical records. The disease course, medication history, C/D ratio, and visual field defects of POAG patients are shown in Table 1 and Supplementary Table S1.

### 2.2. Ophthalmic Examinations and Blood Tests

All study subjects underwent comprehensive ophthalmic examinations, including refraction, best-corrected visual acuity (in logMAR scale), tonometry, slit-lamp biomicroscopy, gonioscopy, ocular biometry, visual field, and optical coherence tomography (OCT). The IOP was measured using Goldmann applanation tonometry (Haag-Streit, Konig, Switzerland). The anterior chamber and lens were examined with slit-lamp biomicroscopy (Haag-Streit model BQ-159 900; Haag-Streit). Non-contact partial coherence interferometry (IOL Master V3.01, Carl Zeiss Meditec AG, Jena, Germany) was employed to measure the axial length (AL), central corneal thickness (CCT), and anterior chamber depth (ACD). The visual field defect was evaluated by the Humphrey MATRIX (Carl Zeiss, Germany), and the retinal nerve fiber layer (RNFL) thickness was measured by the Cirrus HD-OCT 4000 (Carl Zeiss, Germany). All OCT images fulfilled the OSCAR-IB quality control criteria for retinal OCT scans. Fasting peripheral blood samples were collected for routine blood and biochemical tests.

### 2.3. Oxylipins Analysis

The oxylipins analysis was performed by Sensichip Biotechnology Co., Ltd. (Shanghai, China) according to the established procedures. The oxylipins analysis was conducted using the liquid chromatography (LC)/mass spectrometry (MS) platform (Thermo, Ultimate 3000LC, Q Exactive). Chromatographic separation utilized an ACQUITY UPLC HSS T3 column (100 mm × 2.1 mm, 1.8 μm) (Waters Corporation, Milford, MA) with a binary solvent system (solvent A: 0.05% formic acid in water; solvent B: acetonitrile).

The gradient elution program was 0–1 min, 95% A; 1–12 min, 95% A; 12–13.5 min, 5% A; 13.5–13.6 min, 95% A; 13.6–16 min, 95% A. The column temperature was maintained at 40 °C, with a flow rate of 0.3 mL/min and an injection volume of 5 μL. Full-scan mode (m/z range 7–1050) with data-dependent secondary mass spectrometry scanning (TopN = 10) was used, operating in both positive and negative ion modes. The MS parameters were set as follows: heater temperature = 300 °C (+) and 300 °C (−); sheath gas flow rate = 45 arb (+) and 45 arb (−); auxiliary gas flow rate = 15 arb (+) and 15 arb (−); sweep gas flow rate = 1 arb (+) and 1 arb (−); spray voltage = 3000 V (+) and 3200 V (−); capillary temperature = 350 °C (+) and 350 °C (−); and S-Lens RF level = 30% (+) and 60% (−). The compounds were identified based on the retention time, accurate mass, and fragmentation patterns as compared with the authentic standards and database entries (http://metlin.scripps.edu access date on 1 August 2023).

The oxylipins analysis was conducted using SIMCA-P software (V14.1, Sartorius Stedim Data Analytics AB, Umea, Sweden). Principal component analysis (PCA) and orthogonal partial least squares discriminant analysis (OPLS-DA) were applied to determine the differentially abundant oxylipins between the POAG and cataract subjects. In the OPLS-DA permutation test, R^2^ and Q^2^ values indicated the model’s explainability and predictability, respectively. The oxylipins with a variable importance in projection (VIP) score > ±1, *p* < 0.05, fold change (FC) > 1.5 or <0.7, and area under the receiver operating characteristic (ROC) curves (AUC) > 0.7 were considered as the differentially abundant oxylipins. Hierarchical clustering maps and the scatter plots were generated using the ggplot package (v.3.3.0). KEGG (http://www.genome.jp/kegg/ access date on 1 August 2023) and MetaboAnalyst (http://www.metaboanalyst.ca/ access date on 1 August 2023) were utilized in the pathway analysis.

### 2.4. Statistical Analysis

The data were presented as means ± standard deviation (SD). An independent *t*-test was used to analyze the variables with normal distribution, while a non-parametric Mann–Whitney U test was used to analyze the variables not following the normal distribution. Categorical data were analyzed using Fisher’s exact test. A Pearson correlation was performed between the clinical and biochemical parameters and the differentially abundant oxylipins. The AUC was calculated to assess the prediction potential of the differential abundant oxylipins. The sample size was determined by previously published articles. All statistical tests were conducted using IBM SPSS STATISTICS 26 (SPSS Inc., Chicago, IL, USA). *p* < 0.05 was considered statistically significant.

## 3. Results

### 3.1. Demographics and Clinical Examinations of the Study Subjects

The age, sex, height, weight, and body mass index (BMI) of the POAG subjects showed no statistically significant differences as compared to the cataract control subjects (Table 1). For the blood biochemical tests, only the high-density lipoprotein-cholesterol (HDL-C) of POAG patients (1.41 ± 0.29 mmol/L) was significantly lower than that of the control subjects (1.65 ± 0.36 mmol/L, *p* = 0.041).

There were no statistically significant differences in BCVA, AL, CCT, and ACD between the POAG and the cataract subjects (Table 2). The POAG subjects have significantly higher IOP (23.52 ± 8.46 mmHg) than the cataract control subjects (13.53 ± 1.99 mmHg, *p* < 0.001). The retinal and visual field parameters were only recorded in the POAG patients due to the opacity of the refractive medium in the cataract subjects.

### 3.2. Identification of Oxylipins in Aqueous Humor of Primary Open-Angle Glaucoma Subjects

**Figure 1:**
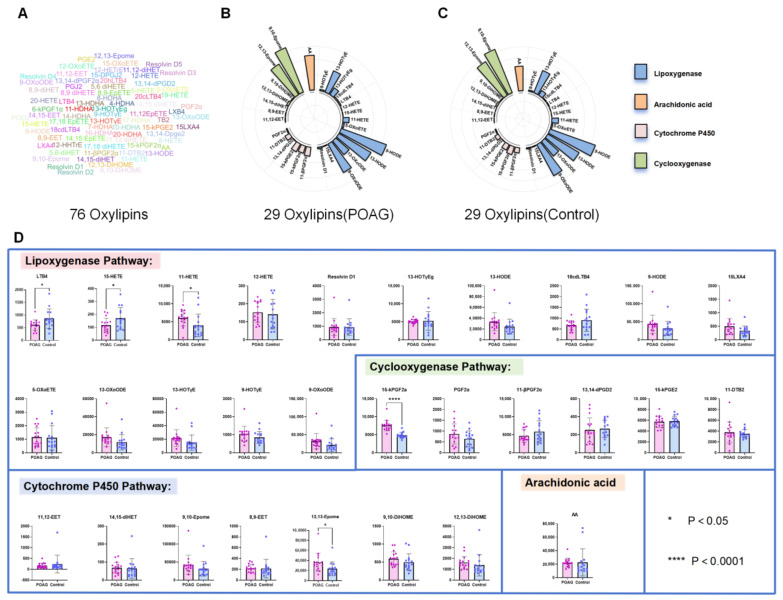
Univariate significance analysis. (**A**) Word Cloud diagram: a total of 76 oxylipins; Grouped Radiographic Histogram: Classification and specific content of 29 oxylipins in the POAG group (**B**) and control (**C**). Blue represents lipoxygenase, orange represents arachidonic acid, pink represents cytochrome P450, and green represents cyclooxygenase. (**D**) Histogram: red represents POAG, blue represents the control group, * *p* < 0.05, **** *p* < 0.0001.

The LC/MS analysis identified a total of 76 oxylipins in the aqueous samples of the POAG and cataract control subjects (Figure 1A), of which 29 reached the detection limit, including arachidonic acid, cyclooxygenase (COX) oxylipins, lipoxygenase (LOX) oxylipins, and cytochrome 450 (CYP450) oxylipins (Figure 1B,C). Compared to the control group, 20 oxylipins, including 15-keto-prostaglandin F2α (15-kPGF2α), 12,13-epoxyoctadecenoic acid (12,13-Epome), and 11-hydroxyeicosatetraenoic acid (11-HETE), were significantly increased, and 9 oxylipins, including leukotriene B4 (LTB4) and 15-hydroxyeicosatetraenoic acid (15-HETE), were significantly decreased in the POAG group (Figure 1D).

**Figure 2:**
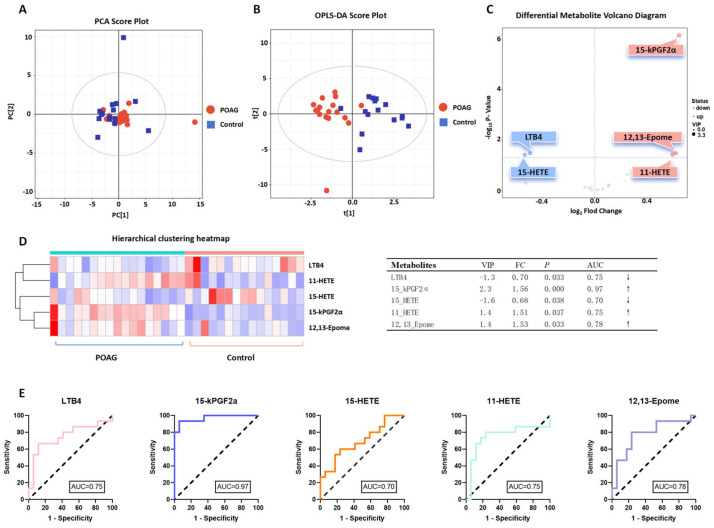
Metabolomic multivariate analysis. (**A**) Principal component analysis (PCA) plots and (**B**) orthogonal projections to latent structures–discriminate analysis (OPLS-DA) score plots illustrating the clustering and dispersion of the two groups. Red represents POAG; blue represents the control group. (**C**) Volcano plot: circles represent each differential oxylipin; red represents up-regulation and blue represents down-regulation. (**D**) Hierarchical clustering heatmap demonstrates the distribution of oxylipins in POAG and control groups. Red represents up-regulation and blue represents down-regulation. (**E**) The ROC curve evaluates the diagnostic performance.

In order to identify the differentially abundant oxylipins, a PCA model (Figure 2A) and an OPLS-DA (Figure 2B) model were established through multivariate analysis. The multivariate analysis identified five oxylipins, 15-kPGF2α (*p* < 0.05, VIP = 2.3, FC = 1.56), 12,13-Epome (*p* < 0.05, VIP = 1.4, FC = 1.53), 11-HETE (*p* < 0.05, VIP = 1.4, FC = 1.51), LTB4 (*p* < 0.05, VIP = −1.3, FC = 0.70), and 15-HETE (*p* < 0.05, VIP = −1.6, FC = 0.68) (Figure 2C,D). The AUC analysis showed that 12,13-Epome (AUC = 0.78, *p* = 0.033), 11-HETE (AUC = 0.75, *p* = 0.037), 15-HETE (AUC = 0.70, *p* = 0.038), and LTB4 (AUC = 0.75, *p* = 0.033) showed good prediction performance, while 15-kPGF2α (AUC = 0.97, *p*< 0.0001) showed excellent prediction performance (Figure 2E).

**Figure 3:**
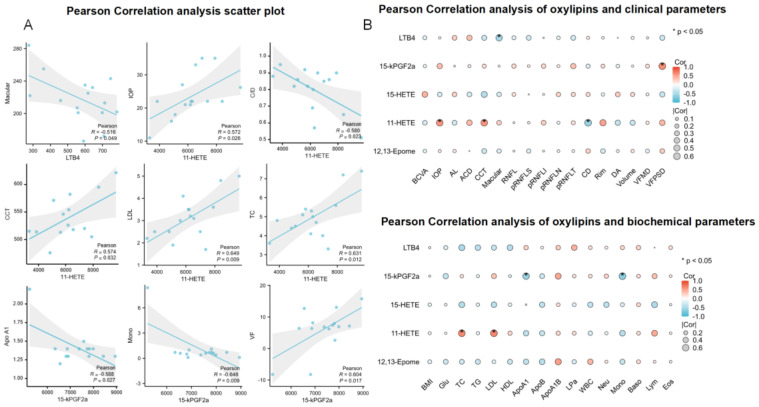
Pearson correlation analysis. (**A**) Scatter plot and (**B**) Pearson correlation analysis of oxylipins and clinical and biochemical parameters: red represents a positive correlation; blue represents a negative correlation. The darker the color, the greater the strength of the relationship, and vice versa. * stands for *p* < 0.05.

We further evaluated the correlation between the five differentially abundant oxylipins and the clinical and biochemical parameters in the POAG subjects. The Pearson correlation showed that LTB4 was moderately and negatively correlated with macular thickness (r = −0.51, *p* < 0.05). Moreover, 11-HETE was moderately and positively correlated with IOP (r = 0.57, *p* < 0.05), CCT (r = 0.57, *p* < 0.05), low-density lipoprotein-cholesterol (LDL-C; r = 0.64, *p* < 0.05), and TC (r = 0.63, *p* < 0.05) and moderately and negatively correlated with C/D ratio (r = −0.58, *p* < 0.05). In addition, 15-kPGF2α was moderately and negatively correlated with Apo A1 (r = −0.58, *p* < 0.05) and mononuclear cells (r = −0.64, *p* < 0.05) and moderately and positively correlated with visual field (r = 0.60, *p* < 0.05) (Figure 3). The Pearson correlation coefficient was interpreted as follows: 0.0 < r < 0.3 (negligible correlation), 0.3 < r < 0.5 (low correlation), 0.5 < r < 0.7 (moderate correlation), 0.7 < r < 0.9 (high correlation), and 0.9 < r < 1.0 (very high correlation).

**Figure 4:**
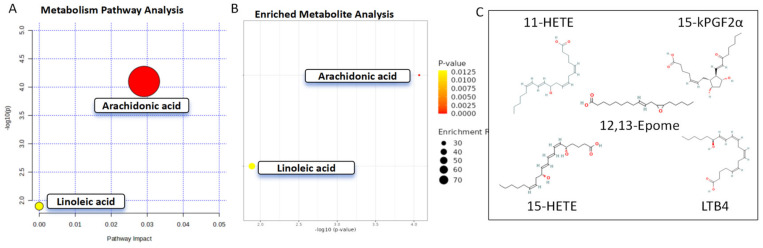
Pathway analysis. (**A**) Bubble plot and (**B**) enriched metabolite analysis indicate enrichment pathways in the AH between POAG and control. (**C**) Molecular structure and oxidation sites of 5 oxylipins.

To clarify the pathways of the differentially abundant oxylipins, the Metaboanalyst and KEGG analyses demonstrated that 15-kPGF2α, 11-HETE, 15-HETE, and LTB4 were enriched in the arachidonic acid pathway, while 12,13-Epome was enriched in the linoleic acid pathway (Figure 4).

## 4. Discussion

The results of this study demonstrated that (1) five differentially abundant oxylipins (15-kPGF2α, LTB4, 12,13-EpOME, 15-HETE, and 11-HETE) were identified in the aqueous humor samples of the POAG subjects; (2) the differentially abundant oxylipins were enriched in the arachidonic acid and linoleic acid pathways; (3) the differentially abundant oxylipins were correlated with the clinical and biochemical parameters in the POAG subjects. Collectively, this study, for the first time, delineated the oxylipin profile in the aqueous humor samples of the POAG patients.

**Figure 5:**
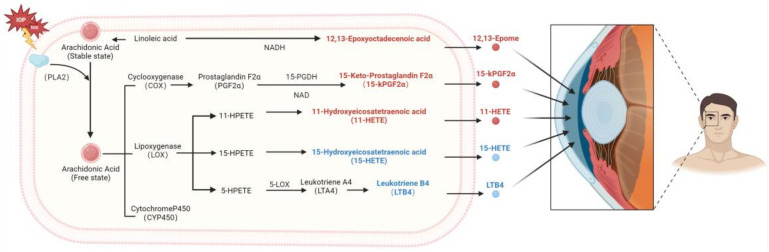
Metabolic pathway profiling. Linoleic acid–arachidonic acid–oxylipins.

Lipids are the essential cellular components, contributing to cell membrane structure, signal transduction, and the regulation of immune inflammation [15]. Oxylipins, the specific lipids mediating oxidative stress and inflammation, reduce the biological activity of the lipids upon oxidation and play significant roles in cardiovascular diseases and neurodegeneration [16]. Previous studies have reported the association between POAG and lipids [5,6,14], and our previous research also identified the dysregulation of lipid metabolism and the changes in oxylipins in the plasma of POAG patients [5,6,13,17]. Aqueous humor, directly involved in IOP regulation, offers direct insights into the metabolic regulations in POAG [18] (Figure 5).

15-kPGF2α is generated from arachidonic acid via the COX enzyme-mediated oxidation of prostaglandin F2α (PGF2α) [19]. PGF2α binds to the FP receptors and activates the Ca^2+^/IP3 pathway [20], regulating pro-inflammation [21] and IOP [22]. PGF2α has been reported as a marker of oxidative stress and inflammation [23]. Research indicates that 15-kPGF2α levels rise significantly in parturition [24]. 15-kPGF2α recruits leukocytes and macrophages via the NFκ-B pathway and promotes cytokine secretion. COX-1 and COX-2 regulate and control 15-kPGF2α levels, with COX-1 active in early inflammation and COX-2 in later stages [24,25]. There is also a reported association between 15-kPGF2α and cardiovascular disease progression [26]. In this study, we identified a negative correlation between 15-kPGF2α and monocytes, suggesting that it may be involved in inflammation regulation. Notably, we have reported 15-kPGF2α as a differentially abundant oxylipin in the plasma of POAG patients [13]. 15-kPGF2α has high AUC values (aqueous humor: AUC = 0.97; plasma: AUC = 0.94), suggesting that 15-kPGF2α could be involved in the development of POAG. However, the mechanisms of 15-kPGF2α in POAG remain unclear and require further investigation.

Leukotriene B4 (LTB4) is produced from arachidonic acid via the 5-lipoxygenase (5-LOX) and 5-lipoxygenase-activating protein (FLAP) complex [27]. LTB4—by binding to the receptors BLT1 or BLT2, chemotaxis, and the activation of neutrophils—promotes the expression of inflammatory factors and is generally considered a potent pro-inflammatory mediator [28]. However, in this study, we found a decrease in LTB4 in the aqueous humor of POAG subjects. The inhibition of LTB4 has been reported to be involved in inflammation alleviation [29,30]. Subbarao et al. [31]. found that in a chronic atherosclerosis model, LTB4/BLT-1 promotes inflammation and atherosclerosis, while 5-LOX inhibition fully prevents these events. This may be due to the initiation of apoptosis in the chronic phase, where reduced 5-LOX activity limits the LTB4 activation of leukocyte Toll/interleukin receptors, preventing organ damage [32,33]. POAG is also chronic and characterized by the apoptosis of RGCs. In addition, the correlation analysis revealed a negative correlation between LTB4 and macular thickness, suggesting that reduced LTB4 levels may be associated with protective mechanisms.

12,13-Epome is produced from linoleic acid oxidation [34]. It induces mitochondrial dysfunction, promotes oncogene expression, and regulates inflammation [35]. Studies have found that 500 μM 12,13-Epome significantly induces mitochondrial dysfunction and cell death [36]. In *Escherichia coli*, the inflammatory response induced by 12,13-Epome decreases with the increase in 12,13-DiHOME [37,38]. Notably, 12,13-DiHOME has also been reported in the aqueous humor samples of POAG patients [17]. In this study, we found an increase in 12,13-Epome (Figure 2C). We previously also identified changes in the linoleic acid and α-linolenic acid pathways in the plasma samples of POAG patients [13], indicating that the 12,13-Epome could be involved in POAG, potentially related to mitochondrial dysfunction, oxidative stress, and inflammation.

15-HETE and 11-HETE are conjugated tetraenoic acids produced from arachidonic acid via LOX enzymes [39,40]. 15-HETE and 11-HETE have pro-inflammatory effects and cause cell damage and pain in arthritis and asthma [41]. In ophthalmic diseases, Ambaw et al. [42] reported that 15-HETE in tears significantly decreased after thermopulsation treatment. In proliferative diabetic retinopathy (PDR), 15-HETE is markedly increased in the retina, enhancing NADPH oxidase expression and ROS generation, which increases vascular permeability. 15-HETE also disrupts VEGF and PEDF balance, promoting inflammation and neovascularization. In this study, we found a decrease in 15-HETE but an increase in 11-HETE. Pearson correlation analysis indicated that 11-HETE is positively correlated with IOP and CCT and negatively correlated with the C/D ratio. Its specific mechanisms require further research investigations.

Previous POAG metabolomics studies have found that amino acid metabolism [18,43], lipid metabolism [13,17,44], and mitochondrial energy metabolism [45] are involved in POAG. In this study, we found that oxylipins are related to the inflammatory pathways, which play important roles in POAG.

There are several limitations in this study. First, due to the lack of a standardized sample size estimation in targeted metabolomics, the sample size in this study was determined based on previous research. Consequently, there may be potential biases and limitations due to the small sample size. Second, disease progression is a dynamic process, and a single measurement may not fully reflect the entire metabolic status of the disease in the patients.

Recent advances in metabolomics, supported by improved detection technologies, analytical platforms, and AI-integrated and multi-omics analysis, have enabled the identification of more metabolites and reliable parameters [46]. This study provides clinically valuable biomarkers and offers new insights into POAG treatment, suggesting that lipid regulation and anti-inflammatory approaches could complement therapies of POAG.

## 5. Conclusions

In summary, this study identified the oxylipins profile in the aqueous humor of POAG patients. The five differentially abundant oxylipins are enriched in the AA and LA pathways, implicating that the inflammation pathway may be involved in POAG.

## Figures and Tables

**Table 1:** Demographics of the POAG and control subjects.

	POAG (*n* = 17)	Control (*n* = 15)	*p*
Age (years)	64.71 ± 10.31	66.47 ± 7.74	0.593 ^a^
Sex (male/female)	15/2	12/3	0.645 ^b^
Height (m)	1.61 ± 0.09	1.64 ± 0.05	0.307^ a^
Weight (kg)	60.90 ± 8.22	64.20 ± 7.86	0.256^ a^
BMI (kg/m^2^)	24.31 ± 2.50	23.51 ± 2.75	0.399^ a^
SBP (mmHg)	137.88 ± 18.97	133.60 ± 15.56	0.494^ a^
DBP (mmHg)	80.00 ± 12.83	85.73 ± 14.31	0.152^ a^
WBC (×10^9^/L)	7.48 ± 2.55	7.03 ± 2.09	0.738 ^c^
Neu (×10^9^/L)	5.08 ± 1.57	4.65 ± 1.90	0.486^ a^
Lym (×10^9^/L)	1.95 ± 0.74	2.27 ± 0.68	0.225^ a^
Mono (×10^9^/L)	1.05 ± 1.91	0.54 ± 0.22	0.317 ^a^
Eos (×10^9^/L)	0.13 ± 0.10	0.20 ± 0.14	0.178^ a^
Baso (×10^9^/L)	0.16 ± 0.24	0.07 ± 0.06	0.147^ a^
Glucose (mmol/L)	6.59 ± 2.02	6.02 ± 0.94	0.992 ^a^
TC (mmol/L)	4.96 ± 1.08	5.52 ± 0.59	0.090 ^a^
TG (mmol/L)	1.18 ± 0.41	1.16 ± 0.50	0.942 ^a^
HDL (mmol/L)	1.41 ± 0.29	1.65 ± 0.36	0.041 ^a^
LDL (mmol/L)	3.14 ± 0.94	3.37 ± 0.71	0.442 ^a^
Apo-A1 (g/L)	1.36 ± 0.27	-	-
Apo-B (g/L)	1.03 ± 0.22	-	-
Apo-A1/Apo-B	0.77 ± 0.14	-	-
LPa (mg/L)	124.58 ± 93.28	-	-

BMI, body mass index; SBP, systolic blood pressure; DBP, diastolic blood pressure; WBC, white blood cell; Neu, neutrophils; Lym, lymphocytes; Mono, mononuclear cells; Eos, eosinophils; Baso, basophilic granulocytes; TC, total cholesterol; TG, triglyceride; HDL, high-density lipoproteincholesterol; LDL, low-density lipoprotein-cholesterol; Apo A-1, Apolipoproteins A-1; Apo B, Apolipoproteins B; LP(α), lipoproteinsα; POAG, primary open-angle glaucoma. Continuous variables are presented as mean ± SD according to the normality of the data. Categorical variables are presented as proportions. Statistical test: ^a^ Student’s *t*-test. ^b^ Fisher’s exact test. ^c^ Mann–Whitney U test.

**Table 2:** Clinical parameters of the POAG and control subjects.

	POAG (*n* = 12)	Control (*n* = 15)	*p*
Laterality (R/L)	6/6	10/5	0.452 ^b^
BCVA (logMAR)	0.65 ± 0.85	0.24 ± 0.24	0.085 ^c^
IOP (mmHg)	23.52 ± 8.46	13.53 ± 1.99	< 0.001 ^a,^*
AL (mm)	23.31 ± 1.05	23.42 ± 0.79	0.751^ a^
CCT (µm)	547.63 ± 46.01	536.46 ± 44.85	0.541^ a^
ACD (mm)	3.10 ± 0.33	3.17 ± 0.27	0.557^ a^
Macular thickness (µm)	237.40 ± 33.25	-	-
RNFL thickness (µm)	58.10 ± 7.99	-	-
C/D	0.78 ± 0.16	-	-
S-pRNFL (µm)	70.20 ± 12.24	-	-
I-pRNFL (µm)	60.50 ± 12.34	-	-
N-pRNFL (µm)	56.10 ± 8.56	-	-
T-pRNFL (µm)	48.40 ± 11.13	-	-
Disk rim Area (mm^2^)	0.69 ± 0.34	-	-
Disk area (mm^2^)	1.95 ± 0.35	-	-
Cup volume (mm^3^)	0.53 ± 0.43	-	-
VF-MD (dB)	−23.18 ± 5.49	-	-
VF-PSD (dB)	8.17 ± 2.16	-	-

* *p* <0.05; ^a^ Student’s *t*-test; ^b^ Fisher’s exact test; ^c^ Mann–Whitney U test; BCVA, best-corrected visual acuity; IOP, intraocular pressure; AL, axial length; CCT, central corneal thickness; ACD, anterior chamber depth; RNFL thickness, retinal nerve fiber layer; C/D, cup/disk ratio; S/I/N/T-pRNFL, superior/inferior/nasal/temporals peripapillary retinal nerve fiber layer; VF-MD, visual field main defect; VF-PSD, visual field pattern standard deviation.

## Data Availability

The original contributions presented in the study are included in the article/Supplementary Materials, further inquiries can be directed to the corresponding authors.

## Supplementary Materials

The following supporting information can be downloaded at: https://www.mdpi.com/article/10.3390/biom14091127/s1, Table S1: Glaucoma medications used in the study subjects.
