# Supplementary material for: Oxylipins in Aqueous Humor of Primary Open-Angle Glaucoma Patients
Source: Biomolecules. 2024 Sep 5;14(9):1127. doi: 10.3390/biom14091127 (PMC11430124; doi:10.3390/biom14091127)
Supplement: Supplementary file 1 [file biomolecules-14-01127-s001.zip › biomolecules-3145760-supplementary.pdf]

Supplementary Table S1| Glaucoma medications used in the study subjects							
Patients	Sex	Age	Laterality	Medications	Disease course	C/D	Visual Field（MD）
POAG-1	female	69	L	Brinzolamide, Carteolol, Brinzolamide timolol	＞2 Years	0.51	-14.04dB
POAG-2	male	58	R	Brinzolamide, Timolol Maleate, Brimonidine Tartrate	＞3 Years	0.65	-18.58dB
POAG-3	male	70	R	Brinzolamide timolol, Bemethoate timolol	＞6 Years	0.82	-19.82dB
POAG-4	male	84	L	Brinzolol Timarol	＞10 Years	/	-27.66dB
POAG-5	male	72	R	Timolol Maleate,	＞1 Years	0.86	-29.45dB
POAG-6	male	71	L	Brinzolamide timolol, Brimonidine tartrate.	＞5 Years	0.92	-28.22dB
POAG-7	male	75	L	Brinzolamide timolol, Brimonidine tartrate.	＞3 Years	0.95	-25.95dB
POAG-8	male	71	L	Brinzolamide timolol, Brimonidine tartrate.	＞1 Years	.90	/
POAG-9	male	73	R	Brinzolamide, Brimonidine tartrate,	＞2 Years	/	-9.30dB
POAG-10	male	42	R	Brinzolamide timolol	＞1 Years	0.88	-29.55dB
POAG-11	male	58	R	Brimonidine Tartrate	＞7 Years	0.57	-30.80dB
POAG-12	male	59	L	Carteolol, Brinzolamide,	＞3 Years	0.86	-13.48dB
POAG-13	male	57	R	Brinzolamide timolol, Brimonidine tartrate.	＞6 Years	0.85	-24.35dB
POAG-14	male	58	L	Brinzolamide timolol, Brimonidine tartrate.	＞1 Years	0.82	-3.24dB

POAG-15	male	53	L	Timolol Maleate,	＞5 Years	0.80	-28.51dB
POAG-16	male	58	R	Carteolol, Brinzolamide,	＞3 Years	0.69	-19.82dB
POAG-17	male	72	L	Brimonidine Tartrate	＞10 Years	0.90	-27.66dB
